# Executive function does not predict coping with symptoms in stable patients with a diagnosis of schizophrenia

**DOI:** 10.1186/1471-244X-8-39

**Published:** 2008-05-29

**Authors:** Maarten Bak, Lydia Krabbendam, Philippe Delespaul, Karola Huistra, Wil Walraven, Jim van Os

**Affiliations:** 1 Dept. Psychiatry and Neuropsychology, South Limburg Mental Health Research and Teaching Network, Euron, Maastricht University, Maastricht, The Netherlands; 2 Prins Claus Mental Health Centre, Sittard, The Netherlands; 3 Division of Psychological Medicine, Institute of Psychiatry, London, UK

## Abstract

**Background:**

Associations between coping with and control over psychotic symptoms were examined using the Maastricht Assessment of Coping Strategies-24, testing the hypothesis that the cognitive domain of executive functioning predicted quality and quantity of coping.

**Methods:**

MACS-24 was administered to 32 individuals with a diagnosis of schizophrenia. For each of 24 symptoms, experience of distress, type of coping and the resulting degree of perceived control were assessed. Coping types were reduced to two contrasting coping categories: symptomatic coping (SC) and non-symptomatic coping (NSC; combining active problem solving, passive illness behaviour, active problem avoiding, and passive problem avoiding). Cognitive functioning was assessed using the GIT (Groninger Intelligence Test), the Zoo map (BADS: Behavioural Assessment of Dysexecutive function), Stroop-test and Trail making.

**Results:**

Cognitive function was not associated with frequency of coping, nor did cognitive function differentially predict SC or NSC. Cognitive function similarly was not associated with symptom distress or level of perceived control over the symptom.

**Conclusion:**

There was no evidence that cognitive function predicts quantity or quality of coping with symptoms in people with a diagnosis of schizophrenia. Variation in the realm of emotion regulation and social cognition may be more predictive of coping with psychotic symptoms.

## Background

How people cope with stress can reduce or amplify the effects of adverse conditions on development of emotional distress, but also on the development of psychiatric disorders [1]. It has been suggested that subjects with a diagnosis of schizophrenia employ a limited repertoire of coping strategies with a preference for emotion-focused strategies [1-5] which are regarded as less effective and associated with worse outcomes [6]. It has been argued that qualitative differences in coping strategies impact on symptom severity, relapse prevention, social adaptation and quality of life in subjects with a psychotic disorder [7-14] and may influence the probability of transition from a prodromal or at risk mental state, to full blown disorder with need for care [15].

According to the transactional analysis theory on coping, stress is generated by the person's appraisal of the personal significance of potentially threatening or harmful events and the success of subsequent attempts to cope with perceived demands on that encounter [16]. For the purpose of this paper, coping is defined as all behavioural, emotional and cognitive strategies the person applies in dealing with stress.

There is little work on the various cognitive processes involved in selection of coping responses. Abilities as inhibitory control, working memory, planning, strategic thinking and attention, grouped together in the category of executive function, enable self-regulatory behaviour in people [17]. Especially executive functions are important in eliciting coping when confronted with distress, correlating with the degree in which a person perceives situational control [18]. Given the fact that individuals with a diagnosis of schizophrenia show deficits in executive cognitive function, it may be hypothesised that cognitive impairment in schizophrenia is associated with less effective coping responses. Previous work in patients with schizophrenia suggest that poor executive function and mnemonic impairments may predict maladaptive coping [19-21], associated with coping preferences characterised by avoiding or ignoring [22-26]. Also, the ability to effectively appraise and think about stressors is negatively associated with cognitive deficits [27], which is a premise of coping and may result in less effective coping responses.

The above studies on coping and cognition in schizophrenia, however, used standard coping scales assessing general coping responses with non-specific stress rather than coping with psychotic symptoms and the associated experience of distress. The Maastricht Assessment of Coping Strategies (MACS) is an instrument designed specifically to assess coping with the symptoms that form part of the diagnosis of schizophrenia [28]. An important difference between MACS and other coping instruments is that the focus of MACS is not on coping in general, but on coping with specific symptoms in the context of a severe psychiatric illness. In this context, coping represents an essential part of the treatment for psychotic disorder. A further difference is that the stressor, the psychotic symptom, is socially stigmatised and that the patient, in order to develop coping, must develop an accurate appraisal of the symptom.

MACS assesses the level of distress associated with psychotic symptoms, and 5 coping domains of active problem solving (APS), passive illness behaviour (PIB), active problem avoiding (APA), passive problem avoiding (PPA), and symptomatic coping behaviour (SCB)[28,29]. Symptomatic coping behaviour is defined as going along with the content of the symptom [28,29]. Effectiveness of coping in MACS is assessed by measuring the level of "feeling in control" or perceived control experienced by the person. This perceived control results from (i) motivated behaviour associated with rewarding situations, (ii) coping mechanisms in stressful situations and (iii) the temperamental ability to switch attention or the propensity to resolve conflicts by selecting to respond only to those stimuli which will lead to reward and inhibit responding to inappropriate or negative stimuli [18,30].

Previous work has shown that SC was negatively associated with perceived control over psychotic experiences and also with an increase in risk of transition from prodromal to clinical state. The other four coping domains, however, were positively associated with perceived control, i.e. can be considered as effective strategies [15,28]. Previously, it has been argued that these four coping domain could be usefully combined into one category of coping, the category of Non-Symptomatic Coping (NSC; for more detail about MACS see previous publications[15,28,31])

It was hypothesised that quantity and quality of coping would be associated with better executive function. Is was thus expected that i) executive dysfunction would be associated with more distress and less perceived control, and ii) that executive dysfunction would be associated with less functional coping (more symptomatic and less non-symptomatic coping).

## Methods

### Sample

Subjects were interviewed as part of the Experience Sampling Method – Maastricht Assessment of Coping Strategies (ESM-MACS) study. All subjects were born in the Netherlands and fluent in Dutch. Thirty-four patients (21 men and 13 women) with a clinical DSM-IV [32] diagnosis of schizophrenia were interviewed by trained interviewers at the level of psychologist with the MACS and the Brief Psychiatric Rating Scale (BPRS), 24 item version [33,34]. Mean age was 37.8 years (SD = 12.3, range 20–68). All subjects received standard psychiatric care in various settings. Half of the sample was living in sheltered housing outside the hospital, whereas the remaining 17 subjects lived alone (n = 8), with partner (n = 1) or with parents (n = 8). One subject had regular employment, 2 subjects were retired and 31 (91%) were receiving disability benefit. Nineteen subjects (62%) had received education above secondary school. All subjects were in a stable phase, with no changes in medication, living situation or service provision during the last six months.

The mean BPRS scores per symptom dimension (adjusted for the number of BPRS items that made up the dimension) were: positive dimension: 1.5 (range 1 – 4), negative dimension: 1.1 (range 1 – 2), depressive dimension: 1.6 (range 1 – 4.8) and excitement dimension: 1.2 (range 1 – 1.8).

The study was approved by Medical Ethics Committee of the Academic Hospital Maastricht. People could only enter the study after given written informed consent. They could leave the study without declaration of any reason.

### Assessment scales

#### Coping and psychopathology (MACS-24)

MACS-24 assesses coping in subjects with a psychotic disorder, focussing specifically on coping with symptoms rather than with situations, and evaluating a range of possible different coping strategies for each symptom dimension.

Symptom dimensions – Five dimensions in subjects with schizophrenia were conceptually derived from work examining the BPRS, PANNS [35,36], PSE [37] and SENS [38]. MACS-24 was designed to cover these five symptom dimensions, with symptom descriptions based on symptom definitions described in work using the BPRS, PANNS, PSE and SENS. A prerequisite for the assessment of coping with symptoms is that subjects have to be aware of their symptoms. Therefore, some cognitive and negative symptoms, like abstract thinking, were excluded as subjects are unable to produce conscious appraisals of these [39,40]. Thus, a total of 24 symptoms were grouped *a priori *in 5 dimensions of (I) positive symptoms (suspiciousness, thought broadcasting, thought influence, grandiosity, magical thinking, passivity experiences, hearing voices and non-verbal hallucinations), (II) negative symptoms (blunted affect, lack of initiative, emotional withdrawal, and self-neglect), (III) depressive symptoms (anxiety, somatic fixation, depressive mood and feelings of guilt, lack of energy, diminished social contact), (IV) cognitive symptoms (poor memory, poor attention and concentration, slowed thinking and chaotic thinking), (V) excitement (hostility and euphoria). MACS-24 was designed to assess coping for each symptom. Individuals are asked to indicate the level of distress they experience in relation to each symptom on a 7-point Likert-scale [28], followed by questions aimed at eliciting how individuals cope with symptom-related distress. Verbatim responses are written down and coping is coded afterwards by the interviewer according to the fourteen coping categories described by Carr [28,29]. People are encouraged to be complete and include all the behavioural, emotional and cognitive actions in response to the symptom and associated distress. This open strategy (coping strategies reported by the person) is used to avoid information bias as a result of providing descriptions of specific coping strategies [41]. After assessing the coping strategies, level of perceived control over the symptom as a result of the combined action of behavioural, emotional and cognitive actions in response to the symptom and associated distress is assessed, providing a measure of the subjective effectiveness of coping.

#### Cognitive tasks

##### Zoo Map (Behavioural Assessment of Dysexecutive Syndrome)

The Zoo Map is a subtest from the BADS, a test battery designed for measurement of executive functions [42-44]. It is designed to assess difficulties due to executive deficits patients encounter in every day life. Subjects are given a map of a zoo and a set of instructions relating to places they have to visit and rules they must follow. In the first part of the test, the subject is required to plan a route which enables them to visit all the places without breaking any rules. In the second part, the order in which places should be visited is specified. The score on the first part of the test was used, as this part involves planning and monitoring of behaviour. The Dutch version of the BADS was used [45]. The test-retest stability of this Dutch version was 0.85 [46].

##### GIT (Groningen Intelligence Test)

The shortened version of the GIT was used as a measure of general ability [47]. The GIT is a test of general intelligence that is used in the Netherlands for purposes comparable to the Wechsler Adult Intelligence Scale (WAIS) [48]. There is general agreement on which subtests are to be used to arrive at good approximation of a full scale IQ [47].

##### Stroop Color Word Test (SCWT)

This test involves selective attention, by displaying colour names, coloured patches and colour names printed in incongruously coloured ink [49]. The amount of extra time needed to discard irrelevant but salient information (verbal) in favour of less obvious aspects (colour naming) is recorded [50]. For the current analysis, the time needed to perform the third task (i.e., reading colour names printed in incongruously coloured ink) was used (hereafter: Stroop interference)

##### Trail Making Test (TMT)

This test is used to measure visual conceptual and visuomotor tracking [51]. The TMT test consists of a part A, which are 25 consecutively numbered circles to be connected. Part B has 13 numbered circles and 13 circles with the letters A through M, again to be connected but now the task requires switching between numbers and letters. The B/A ratio of performance in the TMT provides a measure of executive function [52] (hereafter: TMT interference).

#### Analyses

Of the 34 people who participated, 2 showed incomplete data on MACS and the cognitive battery. Therefore, 32 subjects were used in this report. (See table 1)

**Table 1:** Sample description

	Total	Men (n = 20)	Women (n = 12)
Age (mean years)	35.9 (range 20–68)	31.6 (range 20–60)	44.9 (range 20–68)
Employment status:			
Regular job	15%	16.3%	12.7%
No/Sheltered work	85%	83.7%	87.3%
Social:			
Alone	24%	26.5%	18.8%
Partner	2%	0.0%	5.8%
Family/parents	33%	38.9%	20.9%
Sheltered living	41%	34.9%	55.0%
Level of education:			
Primary school	22%	22.5%	20.3%
Secondary school	20%	21.1%	15.9%
Lower & Intermediate vocational education	43%	38.1%	52.2%
Higher vocational education & University	35%	18.4%	11.6%

##### Symptom level

The data were analysed with STATA, version 9.2 [53]. A data file was constructed in which the 32 patients included in the study contributed 768 observations: one for each of the 24 symptoms described above (32 × 24 = 768 symptom observations). Each symptom pertained to one of the 5 symptom dimensions as described above. In the symptom-level analyses, dependent variables were level of distress and level of control, and independent variables were symptom dimensions and cognitive variables. According to the hypotheses specified above, executive dysfunction would be associated with more distress and less perceived control.

##### Coping observation level

A data file was constructed where for each symptom the scores of each of 14 coping strategies were scored, resulting in a data file with 32 (subjects) × 24 (symptoms) × 14 (coping strategies) = 10752 observations. Each coping strategy pertained to one of 5 broader coping domains as described above, which was further simplified to the two domains of symptomatic and non-symptomatic coping as described earlier. In the coping level analyses, the binary coping variable (presence or absence of coping for the specific symptom and coping category) was the dependent variable, and coping type, cognition, as well as their interactions, were dependent variables. The interaction between coping type and cognition was fitted as according to the hypotheses specified above the impact of cognitive impairment on quantity of coping would be evident for functional coping, i.e. NSC, in particular.

As observations were clustered within individuals for both data sets (symptom level and coping level), multilevel random regression analyses were conducted using the STATA XTGEE (binary coping variable) and XTREG (continuous distress and control variables) routines. For the coping level analyses, effect sizes were expressed as odds ratios (OR) with their 95% confidence interval (95%CI), whilst for the symptom level analyses effect sizes were expressed regression coefficients (B). Model contributions of independent variables and interactions were assessed by Wald test and, in the case of significant interaction, stratified effect sizes were calculated as linear combinations of main effects and interactions.

## Results

### Symptom level

The mean BPRS score was 31.7 (SD = 6.0, range 24 – 51). Twenty one (66%) subjects were in symptomatic remission (i.e. meeting the psychopathology criterion but not the time criterion of remission) as defined recently [54,55]. However, symptomatic remission involves only six of 24 BPRS items and does not exclude residual-level symptomatology as typically seen in chronic patients. Thus, all subjects had at least one symptom present per MACS interview. The mean number of MACS symptoms per subject was 7.0 (SD = 4.0, range 1 – 17) with a mean distress score of 4.2 (SD = 1.9, range 1–7) and a mean level of perceived control of 4.2 (SD = 1.9 range 1–7). The mean number of MACS symptoms per subject was 7.0 (SD = 4.0, range 1 – 17) with a mean distress score of 4.2 (SD = 1.9, range 1–7) and a mean level of perceived control of 4.2 (SD = 1.9, range 1–7).

The mean IQ score was 101 (SD = 15.8, range 72 – 128), the mean TMT interference score was 143 (SD = 402, range -4 – 2315), the mean Stroop interference score was 126 (SD = 91, range 16 – 350) and the mean Zoo Map score was 3.59 (SD 3.00, range 0 – 8).

### Associations between distress, control and cognitive impairment

Apart from a weak negative association between experience of distress and IQ (B = -0.04; 95% CI: -0.06 – -0.01), no associations were apparent with the other three cognitive tests of TMT interference, STROOP interference and Zoo Map. Perceived control was similarly not associated with any of the four cognitive variables (See table 2).

**Table 2:** Associations between 4 cognitive variables and symptom-related distress (distress experience), perceived control as the result of coping (perceived control), and frequency of symptomatic (symptomatic coping) and non-symptomatic coping (non-symptomatic coping) to reduce distress

	IQ		TMT interference		Stroop interference		ZOO MAP	
	B	95%CI	B	95%CI	B	95%CI	B	95%CI
Distress experience	- 0.04	-0.06 - -0.12*	-0.05	-0.16 – 0.05	0.79	-0.23 – 1.82	0.75	-0.08 – 0.23
Perceived control	0.03	-0.00 – 0.05	-0.01	-0.09 – 0.11	-0.17	-1.20 – 0.85	0.03	-0.14 – 0.19

* p < 0.05

#### Coping level

There were 373 instances of coping with the following distribution: active problem solving (APS): 22%, passive illness behaviour (PIB): 17%, active problem avoiding (APA): 15%, passive problem avoiding (PPA): 19% and symptomatic coping behaviour: 27%, indicating that the group of Non-Symptomatic coping made up 73% of all coping. (Table 3 displays the frequency per coping domain). Instances of coping were distributed across patients, only a single patient had no instances of coping at all.

**Table 3:** Frequency of coping per coping domain

Coping domain	Coping strategy	frequency	%
Active Problem Solving (APS)	Distraction	24	10.5
	Problem solving	51	22.3
	Help seeking	8	3.5

Passive Illness Behaviour (PIB)	Physical change	48	21.1
	Prescribed medication	9	3.9
	Non-prescribed medication, drugs	8	3.5

Active Problem Avoiding (APA)	Socialisation	10	4.4
	Shifted attention	31	13.6
	Task performing	6	2.6
	Indulgence	9	3.9

Passive Problem Avoiding (PPA)	Non-specific activities	29	12.7
	Isolation	22	9.7
	Suppression	19	8.3

Symptomatic	Symptomatic behaviour	99	43.4

Total		373	11.7

### Associations with cognitive impairment

None of the four cognitive variables predicted coping behaviour (IQ: OR = 1.01, 95% CI = 0.99 – 1.04; Trail-making: OR = 0.99; 95% CI = 0.92 – 1.08; Zoo Map: OR = 1.07, 95% CI = 0.91 – 1.25; Stroop: OR = 0.54; 95% CI = 0.21 – 1.39). There was no evidence that the association between cognition and coping was moderated by type of coping (i.e. symptomatic or non-symptomatic) for Trail-making, Zoo Map and IQ (p = 0.43, p = 0.69 and p = 0.051 respectively). Although there was evidence for such an interaction for Stroop (p = 0.034), stratified analyses revealed that the association with coping was non-significant for both non-symptomatic (OR = 1.32, 95% CI = 0.88, 1.99) and symptomatic coping (OR = 0.56, 95% CI = 0.27–1.16).

## Discussion

It was hypothesised that quantity and quality of coping would be positively associated with executive function; in particular that cognitive impairment would be associated with more distress and less perceived control, as well as with less functional coping in people with a diagnosis of schizophrenia. However, with the exception of an association with IQ, which might have been arisen by chance, there was no evidence that executive dysfunction was associated with quality or quantity of coping.

The explanation for this finding may be related to the characteristics of the MACS. The MACS differs from previous coping assessments in four ways: (i) the MACS examines coping with specific psychopathological symptoms and associated distress rather than general coping strategies, and (ii) the MACS focuses on descriptions of self-initiated coping, whereas in conventional coping assessments discrepancies may arise between coping strategies listed in the instrument and coping strategies that are actually used as someone may use coping strategies without recognising these as such [41], (iii) MACS assesses coping only if patients are aware of symptoms and symptom-related distress, as subjects need to recognise a symptom and the associated distress experience to undertake self-protective actions, (iv) perceived control is the outcome variable of effectiveness of coping, that is the result of a self-reported evaluation of the various coping strategies used. MACS does not require insight, but awareness of the experience, similar to an interview with any instrument assessing psychopathology such as PANSS and BPRS. MACS additionally requires that the patient is able to reflect to a certain degree on what effects the symptom has on him/her but for this no insight is required. Therefore, in the MACS, unconscious coping strategies or so-called "automatised" coping strategies people use in every day life are excluded [56,57], as these are not considered true coping strategies according to the definition used for the purpose of these analyses.

It may be argued that this latter characteristic of the MACS is relevant to the lack of any association with cognition in the current study, as reduced awareness, to the degree that it may be associated with poor insight, may be associated with neurocognitive dysfunction, especially poorer executive function [58]. Therefore, the sample may have been selected towards patients with awareness of symptoms and better cognitive function. However, comparing the neurocognitive findings with those of patients and controls in previous studies in this area revealed that mean performance in the current sample was comparable to findings in chronic patients with schizophrenia and substantially lower than performance in control subjects. This suggests that the sample was not selected towards better cognitive functioning.

It has been argued, contrary to what is generally assumed, that the pathway from stress to psychosis is not mediated by cognitive impairment. Rather, there may be two independent pathways, the first being characterised by cognitive impairment and the second by abnormal affective processes [59,60]. For example, it has been demonstrated that the emotional reactivity to stress in the daily lives of patients with schizophrenia is not the consequence of cognitive impairment per se [61]. Elevated emotional reactivity to stress was found in subjects vulnerable to psychosis, suggesting that affective responses in the flow of daily life are an indicator of genetic but also environmental liability to psychosis [60]. The affective state appears a strong predictor of coping function [17,62], with negative affect being associated with maladaptive coping [8] and variation in emotional reactions to stressors being associated with coping dynamics [56,63,64]. Stress, in particular states of negative affect, influences the efficiency of attentional processes and the direction of selective attention. The coping strategy chosen is likely to guide subsequent selection, such that the coping strategy and selective attention are dynamically and reciprocally related [65].

Given the robust null finding with cognitive test variables, there is little to suggest type II error. Nevertheless, the MACS is a new instrument, which has not been used very often and therefore may hold unknown insensitivities with regard to cognitive associations. However, the focus of MACS on authentic report of coping strategies comes closest towards the original definition of coping [16]; it does not incorporate information bias on coping strategies, which is a problem for most other coping instruments. Instead, it emphasises the fact that coping is a conscious activity. As a result of this strict definition of coping, only subjects who are aware of the symptom and concomitant distress are able to reflect, in terms of coping, on their symptoms and distress experience. This conservative definition excludes important areas of experience. Further research with the MACS may contribute to a better understanding of the specific qualities and pitfalls of the MACS in coping research.

The majority of the sample was in a state of remission, but patients in symptomatic remission may display coping for residual symptoms. Thus, the analyses suggested that instances of coping were distributed across patients as only a single patient had no instances of coping at all. The fact that the majority of the sample was in remission cannot be taken to indicate that coping is not relevant in such patients; research suggests that coping strategies are typically used in chronic patients with a degree of residual symptoms, similar to the group in this study [66], and that enhancing coping skills in this group may contribute to better outcomes [67]. Although the mean BPRS score was low, it does not follow that executive function will be better and associations with coping obscured, given the fact that cognitive dysfunction is a stable feature that is only weakly associated with symptomatology [68,69]. It has been suggested that perceived control is the product of motivated behaviour and emotion regulation capacities [18]. In patients with a diagnosis of schizophrenia, coping and perceived control are apparently entirely emotion regulated rather than guided by executive functions [18].

Therefore, this report disputes the association between coping with symptoms and cognitive function, suggesting that quantity and quality of coping with symptoms is part of the affective pathway, independent from cognitive function.

## Limitations

The results of this study should be interpreted in the light of the following limitations.

(i) The number of subjects was limited and therefore the results should be seen as indications for future study rather than conclusive findings; (ii) The group was relatively homogenous. Therefore, differences in cognitive functioning present in subgroups like first-episode patients versus chronic patients or inpatients versus outpatients remained unresolved with regard to differential impact on coping properties and interactions with cognitive functioning. (iii)

The mental effort people with a diagnosis of schizophrenia exhibit in test assessment, especially neurocognitive batteries, is limited [70]. We did not control for this problem in our sample. (iv) While our *a priori *hypothesis focussed on executive function, executive function is only one of the domains of cognitive functioning. Therefore one cannot extrapolate the results to general cognitive dysfunction and coping in people with a diagnosis of schizophrenia. (v) The test that was used to measure intelligence (GIT) was an older version. Therefore, the normative data for this test tend to gave an overestimation of IQ compared to other more recent intelligence tests (i.e. the mean IQ of 101 we reported in this sample, would be comparable with 93). This suggests we studied a representative sample of chronic patients with a diagnosis of schizophrenia. (vi) All subject were on antipsychotic medication. We did not control for the effect of antipsychotic medication, given the small sample size and the fact that the direction of association between cognition and antipsychotic medication is not consistent. (vii) We cannot exclude, on the basis of the current data, that those with better cognitive functioning are better able to comply with MACS procedures, in particular with regard to making accurate appraisals of symptoms. (viii) Although it may be argued that MACS requires that the patient is able to reflect to a certain degree on what effects the symptom has on him/her, this is not the same as requiring insight into illness or the pathological nature of symptoms. (ix) Finally, as mentioned in the discussion, the sample was characterised by stable patients with few symptoms. We cannot exclude that coping with weak symptoms requires relatively little cognitive effort so that associations between executive function and coping in the sample would remain too low to be detected. Therefore, replication in a sample with more severe symptoms is required.

## Conclusion

No evidence was found that coping in patients with a diagnosis of schizophrenia was associated with cognitive function. According the dynamic aspects of coping, use of coping strategies are more likely to be emotion regulated.

## Competing interests

The authors declare that they have no competing interests.

## Authors' contributions

MB designed the study, performed the statistical analyses and wrote the first drafts of the paper, LK was responsible for incorporating cognitive tests, helped in the statistical analyses especially with respect to the cognitive tests and participated in the final drafts, PD was involved in the original design of the study, and participated in the final drafts of this paper, KH interviewed patients and took care of the database, WW refined the original design and interviewed patients, JvO supervised and participated in the analyses and the final draft of this paper.

## Pre-publication history

The pre-publication history for this paper can be accessed here:


